# Chlorophyll *a* Fluorescence and Freezing Tests as Selection Methods for Growth Cessation and Increased Winter Survival in ×*Festulolium*

**DOI:** 10.3389/fpls.2018.01200

**Published:** 2018-08-20

**Authors:** Liv Østrem, Marcin Rapacz, Arild Larsen, Petter Marum, Odd A. Rognli

**Affiliations:** ^1^Department of Grassland and Livestock, Norwegian Institute of Bioeconomy Research (NIBIO), Hellevik i Fjaler, Norway; ^2^Department of Plant Physiology, University of Agriculture in Krakow, Krakow, Poland; ^3^Graminor Ltd., Ridabu, Norway; ^4^Department of Plant Sciences, Faculty of Biosciences, Norwegian University of Life Sciences, Ås, Norway

**Keywords:** breeding, dry matter yield, leaf growth, NDVI, photosynthetic parameters, winter damage

## Abstract

In a ×*Festulolium* population (FuRs0357) of parental origin *Lolium perenne* × *Festuca pratensis*, selection of freezing tolerance by freezing tests on whole plants (FT) and chlorophyll *a* (Chl-*a*) fluorimetry on frozen detached leaves (CF) was assessed in high and low directions during two cycles of selection. The original population went through two cycles of random mating. All selections and non-selected intercrossed generations of the original population were established in field trials at a coastal site and a continental site in Norway. At the coastal site, analyses of Chl-*a* fluorimetry parameters and leaf growth on individual plants in autumn and winter hardiness observed in field plots in spring showed that the first-generation selections for high freezing tolerance were associated with winter hardiness and early growth cessation. The second-generation FT-selections for high freezing tolerance were also associated with winter hardiness, whereas the CF-high selections diverged toward high photosynthetic activity. Both low selections were correlated with high photosynthetic activity. There were smaller variations between generations in unselected generations of the original population. Low accumulated leaf growth and early growth cessation were observed in the second-generation FT-selection for high freezing tolerance, whereas high normalized difference vegetation index (NDVI) were seen in Chl-*a* selections. Both selection methods distinguished diverging selections with significantly different high and low freezing tolerance, but selection efficiency was comparable only for the first selection cycle. Moreover, due to mixed ploidy level in the original population, selection by FT and CF generated diploid and tetraploid plants, respectively, which intensified the response of selection, particularly in the diploid selections. Total dry matter yield (DMY) (mean of three annual cuts for 3 years) of the FT-high selections was lower than for the CF-selections. At coastal sites, selection intensity using freezing tests on whole plants should be adapted to actual climate conditions, to obtain genotypes that balance photosynthetic activity during autumn and good winter hardiness, making them persistent and high yielding.

## Introduction

Predicted effects of future climate changes are increased temperature, longer growing seasons, higher precipitation and more irregular winter climates for higher latitudes (IPCC, 2013; Hanssen-Bauer et al., 2015). Increased autumn temperature entails a temperature–photoperiod interaction, which may impose a trade-off between extended autumn growth and cold acclimation/freezing tolerance of perennial grasses at higher latitudes. In most conditions, freezing tolerance is the main component of the complex winter-hardiness trait (Levitt, 1980). It is maximized through the process of cold acclimation after exposure to low, non-lethal temperatures, and the light energy transformed through photosynthesis (Østrem et al., 2014). Moreover, freezing tolerance, avoidance of cold-induced photoinhibition, and growth cessation, i.e., reduced leaf elongation growth rate and biomass production, seem to be closely interrelated (Hüner et al., 2013; Østrem et al., 2014; Rapacz et al., 2014).

In the Nordic region, sufficient freezing tolerance is crucial for further expansion of the cultivation area of non-native grass species such as *Lolium perenne* L. and ×*Festulolium* (Østrem et al., 2007, 2014). Growth cessation is a pre-condition to successful acclimation to cold (Huner et al., 1993) and, at higher latitudes, is the major reason for inferior winter hardiness of non-native species. Environmental factors such as shorter photoperiod and decreasing temperature signaling the onset of winter are not sensed by non-native plants, resulting in erratic winter survival in temperate climates (Rognli, 2013). For breeding purposes, efficient selection for freezing tolerance is crucial, since selection under natural conditions is time-consuming and expensive, and does not always differentiate sufficiently between genotypes.

Artificial freezing tests for forage grasses have been developed, mainly short-term freeze tests (lethal temperature for 50% plant kill, LT_50_) (Pulli et al., 1996). These show high correlations between relative scores and field survival (*r* = 0.7–0.8) for perennial grasses (Larsen, 1989). However, the correlation between winter hardiness and freezing tolerance of plants cold-acclimated in the laboratory is weaker than for plants cold-acclimated in the field (Gusta et al., 2001; Rapacz et al., 2015a).

Schreiber and Bilger (1993) found chlorophyll *a* (Chl*-a*) fluorimetry to be a relatively sensitive indicator of stress effects on photosynthesis and thus an indirect screening method providing large amounts of precise data related to photosynthesis without damaging the plant (Rapacz and Woźniczka, 2009). Performance index (PI) is the ratio between structural and functional PSII events leading to electron transport within photosynthesis and the energy dissipated or lost from photosynthesis (Clark et al., 2000). It can be calculated based on the absorbed light energy (PI_ABS_). Performance index has been shown to be very sensitive to different stresses and to differ between species (Stirbet and Govindjee, 2011). It can also be used as an indirect indicator of freezing tolerance (see Gonçalves and dos Santos, 2005). In winter wheat, PI_ABS_ can differentiate cultivars with contrasting freezing tolerance when measured both before and after freezing (Rapacz, 2007). It is therefore useful for screening purposes (Rapacz and Woźniczka, 2009).

Normalized difference vegetation index (NDVI), developed by Rouse et al. (1974), is another indirect screening method for indicating the chlorophyll content of vegetation. It is based on differences in leaf absorbance in the red spectrum due to chlorophyll pigments and reflectance in the infrared spectrum caused by leaf cellular structure (Ólafsdóttir and Óskarsson, 2014). It is used as a proxy for growth cessation, but it is very sensitive to ambient temperature conditions (Helgadóttir and Kristjánsdóttir, 2013; Østrem et al., 2015). Thus, NDVI may be a more useful tool when used together with other methods.

To improve winter survival of non-native species, fast and cost-effective screening methods are required for early direct selection for growth cessation or for indirect selection for traits genetically or physiologically related to this trait. The aim of this study was thus to compare the selection efficiency for freezing tolerance of: (i) direct selection using freezing tests of whole plants and (ii) indirect selection using Chl-*a* fluorimetry on frozen detached leaves. An additional aim was to investigate the relationship between winter survival and dry matter yield (DMY) of selections produced by the two methods under field conditions and Chl-*a* fluorimetry parameters on individual plants from the same selections during autumn. To test these screening methods, divergent selections in high and low directions were generated from a backcrossed ×*Festulolium* population (FuRs0357) of *L. perenne* ×*Festuca pratensis* parental origin. The hypotheses were: (1) Direct selection by freezing tests gives a stronger selection response than indirect selection based on photoacclimation (Chl-*a* fluorimetry); (2) selection for high freezing tolerance using direct freezing tests leads to earlier growth cessation than selection based on chlorophyll fluorescence (F_v_/F_m_); and (3) direct selection correlates better to field performance than indirect selection.

## Materials and Methods

### Plant Material and Selection Procedures

**FIGURE 1:**
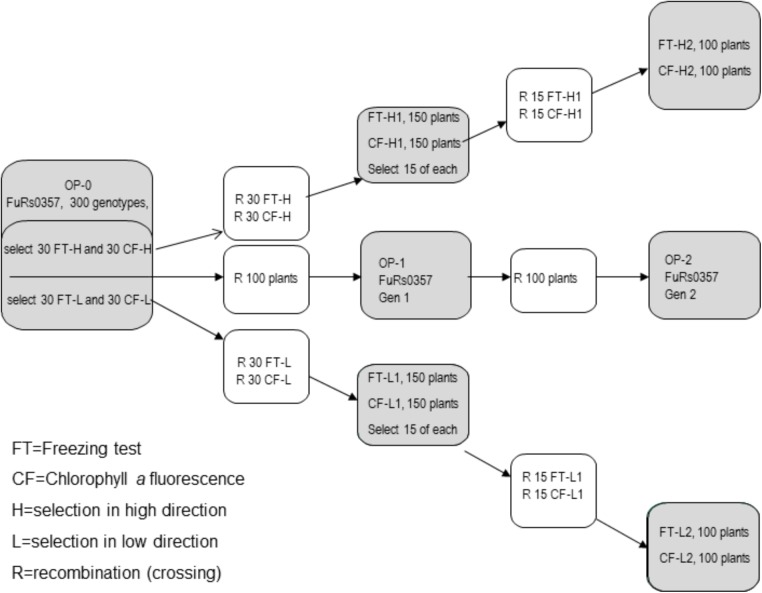
Selection for freezing tolerance by means of a freezing test (FT) and chlorophyll fluorescence (CF) in a high (H) or low (L) direction and recombination (R) in the first or second generations.

The ×*Festulolium* population FuRs0357 (*L. perenne* ×*F. pratensis*) used in this study was expected to be diploid, based on the genome constitution of the genotypes used in the crossings. However, as reported by Bartoš et al. (2011), the population also contains tetraploid genotypes. In 2007, 300 randomly selected genotypes of the original seed lot (OP-0) of the population were established at the Norwegian University of Life Sciences (NMBU), Ås, Norway. Each genotype was cloned into five ramets; one ramet was transferred to NIBIO, Vågønes, Bodø, Norway, for testing of freezing tolerance, one ramet was transferred to the University of Agriculture in Krakow, Poland, for Chl-*a* fluorimetry measurements and three ramets were vernalized at NMBU for intercrossing of selections. The following divergent selections were developed using a selection intensity of 10% (30 genotypes/selection): first-generation selections for high and low freezing tolerance using freezing tests on whole plants (FT-H1 and FT-L1, respectively) and first-generation selections for high and low freezing tolerance using Chl-*a* fluorescence (CF-H1 and CF-L1, respectively). In addition, 100 genotypes were randomly selected among the 300 vernalized genotypes from OP-0 for subsequent intercrossing in isolation to represent unselected synthetic generations (**Figure 1**).

In summer 2008, the 30 genotypes of each of the four selections and the 100 genotypes representing the unselected synthetic OP-0 were isolated in separate pollen-proof greenhouse compartments at NMBU and allowed to intercross by open pollination. Seeds were harvested separately on each plant, and 10 seeds from each of the 30 mother plants per selection were randomly selected and bulked to constitute the first generation of the four selections. A balanced OP-1 was constructed by bulking equal amounts of seeds of each of the 100 unselected OP-0 genotypes.

The number of genotypes used in the first selection cycle was halved, i.e., 150 genotypes of each of the first-generation selections were established, cloned and subjected to a second round of selection by whole-plant freezing tests and Chl-*a* fluorescence measurements with the same selection intensity (10%, i.e., 15 genotypes/selection). Vernalized ramets of the 15 genotypes selected from each first-generation selection and 100 randomly selected genotypes from the OP-1 unselected population were isolated in separate pollen-proof greenhouse compartments and allowed to intercross by open pollination. The second generation selections (FT-H2, FT-L2, CF-H2, and CF-L2) and the unselected OP-2 generation were constructed in the same way as the first-generation selections, i.e., 10 seeds were taken from each of the 15 mother plants per selection, and equal amounts of seeds from each of the 100 OP-1 mother plants were bulked.

#### Description of Freezing-Test Based Selection (FT)

Ramets from 300 genotypes were planted in a greenhouse for tiller production in late October 2007 at NIBIO, Vågønes, Bodø. From mid-January 2008, each genotype was divided into ramets and planted in a peat-sand mixture for freeze testing. Six replicates comprising one ramet per genotype were established. In each replicate, the plants were placed in five boxes each containing 60 genotypes according to Aastveit (1977). After 4 weeks of growth, two replicates were placed in Conviron chambers (E10) for hardening; first 1 week of pre-hardening at 12/6°C day/night and 12 h photoperiod, followed by 2 weeks at 1°C and 16-h photoperiod at a light intensity of PAR = 110 μmol m^-2^ s^-1^. To ensure equal conditions, the position of the boxes was changed twice a week. For freeze testing, the boxes were transferred to a programmable freezer. The temperature was stabilized at -2°C for 24 h, gradually decreased to a pre-determined temperature which was kept constant for 24 h and then gradually raised to 1°C (Pulli et al., 1996). The test temperature ranged from -9 to -12°C for the various replicates. After thawing, the plants were cut to a height of 5 cm above the soil surface and placed under growing conditions in a greenhouse. Freezing tolerance was scored after 2 and 3 weeks of regrowth on individual plants according to a visual scale of 0–9 (0 = dead, 9 = no visible injury) (Larsen, 1978). Plants for the second round of selection, i.e., 150 genotypes from each of the first-generation high and low freezing tolerance selections, were multiplied during winter 2009, and freezing tolerance tests were conducted as described for the first-generation selections. To ensure optimal variation within groups, replicates of high freezing tolerance selections were subjected to some lower test temperatures than replicates of the low freezing tolerance selections (Larsen, 1979).

#### Description of the Chlorophyll Fluorescence-Based Selection (CF)

Upon arrival in Krakow, each plant was divided into four ramets. During the first generation of selection, plants were established in boxes (35 × 75 cm, 15 plants/box) in the universal soil mixture Sterlux (Hollas, Paslek, Poland). After planting, the ramets were grown in an air-conditioned greenhouse for 4 weeks at +15°C (10/14 h, day, night) and light intensity and daylength were supplemented with HPS lamps Agro (Philips) for 300 μmol m^-2^ s^-1^. After this period, the plants were cold-acclimated at 4/2°C (day/night, 10/14 h, PAR = 250 μmol m^-2^ s^-1^, FPS lamps Agro, Philips). Samples for measurements were taken independently on four occasions with a two-day break at days 21 and 24 of cold acclimation (four independent series). In each experimental series, at least 10 leaves were collected in polyethylene plastic bags with string closures and ice was added to ensure nucleation. The freezing-thawing cycle was performed in a programmed freezer, where the temperature was initially decreased from 0 to -15°C (twice) and to -12°C (twice) at a rate of 2°C/h. At the test temperature, samples were maintained for 6 h followed by a temperature increase at a rate of 3°C/h to +2°C. The leaves were stored at this temperature until measurement.

The measurements of Chl-*a* fluorescence induction kinetics were performed on dark-adapted leaves (30 min) using a HandyPea fluorimeter (Hansatech, Kings Lynn, United kingdom). The light pulse intensity was set to 3,000 μmol m^-2^ s^-1^, pulse duration of 1.0 s and fixed gain (1 × ) as described by Rapacz (2007). Fluorescence measurements were made in 10 replicates for each series. Although complete OJIP analysis is performed by calculation of many parameters (Strasser et al., 2000; Rapacz et al., 2015b), only PI_ABS_ was used for selection of plants with the largest variation between clones as regards freezing tolerance of the photosynthetic apparatus (data not shown).

The first round of selection was performed during winter 2007/08 and the second round during winter 2008/09. In the latter case, a storage period of 14 days at 10°C in darkness before planting was added, in order to deplete reserves.

#### Seed Propagation of Selections

Seeds of the first-generation selections (FT-H1, CF-H1, FT-L1, CF-L1, OP-1) were from field plots spaced at sufficient distance to prevent cross-pollination at the Vollebekk Experimental Farm, NMBU, Ås (59°41′N 10°45′E). Seeds of the second generation selections (FT-H2, CF-H2, FT-L2, CF-L2, and OP-2) were produced from isolated plots in a rye field at Staur Experimental Farm (61°25′N 09°57′E). Seeds were harvested for two consecutive years (2011 and 2012), and all seed lots were cleaned at Graminor Ltd., Bjørke, Norway.

### Field Trials With Selections

Field trials with all selections were established in spring 2012 at NIBIO, Fureneset (61°18′N, 5°21′E, 10 m a.s.l.; coastal climate) and Bjørke Experimental Farm (Graminor Ltd, Bjørke, Norway) (61°22′N, 20°42′E, 190 m a.s.l.; continental climate). A complete randomized design with plot size 10.5 m^2^ and three replicates was used for assessment of seasonal biomass production (Fureneset and Bjørke) and winter survival (Fureneset). In the three ley years, the trials were fertilized according to local norms, receiving in total 295 (Fureneset) and 216 (Bjørke) N ha^-1^ year^-1^. At Fureneset, winter damage was calculated according to the formula: [(Previous autumn ground cover - Spring ground cover)/Previous autumn ground cover) × 100]. The percentage spring ground cover was determined when the grass had started growing, on 22 April 2014 and 23 April 2015. Spring cuts and two regrowth cuts were harvested using a Haldrup forage plot harvester. Herbage samples were dried at 60°C for 48 h for estimation of dry matter yield (DMY).

### Autumn Measurements of Leaf Elongation and Chl-*a* Fluorimetry

In mid-May 2013, 30 individual plants were taken from six selections in the field trial at Fureneset and in late June 2014 18 individual plants of all selections in that trial. The plants were established outdoors in large pots, fertilized, watered if necessary and cut during the summer to obtain the same developmental stage as if they were in field. From early September until late November in both years, weekly measurements of leaf elongation were performed on pre-determined leaves and Chl-*a* fluorescence measurements were performed on the same individual plant.

Chl-*a* fluorescence characteristics were studied using the same equipment as described for the Chl-*a* fluorescence-based selections and with the same instrument settings. Chl-*a* fluorescence transient measurements followed by OJIP test analysis are a sensitive and complex method for analyzing the photosynthetic performance of plants (Strasser and Tsimilli-Michael, 2001). Parameters of the OJIP test used for the analyses were calculated as described by Rapacz et al. (2015b), i.e., absorbed energy flux (ABS) per single, Q_A_-reducing PSII reaction center (RC) and phenomenological flux per leaf cross-section (CS) (ABS/CS, ABS/RC, respectively); trapped energy flux (TR_o_) ratios (TR_o_/CS, TR_o_/RC); electron transport flux (ET_o_) ratios (ET_o_/CS, ET_o_/RC); and dissipated energy flux (DI_o_) ratios (DI_o_/CS, DI_o_/RC). PSII performance indices calculated included: PI in relaxed and excited state (PI_CSo_ and PI_CSm_, respectively) on a CS basis (PI_CS_) and performance index calculated on an absorption basis (PI_ABS_). The following yield ratios were also computed: the probability (at *t* = 0) of electron transport beyond Q_A_^-^ (ψ_o_), quantum yield of electron transport (at *t* = 0) (φ_Eo_) and maximum quantum yield of primary photochemistry (at *t* = 0) (F_v_/F_m_). For detailed calculations used, see Rapacz et al. (2015b).

### Normalized Difference Vegetation Index (NDVI)

Ground-based NDVI measurements were made in the field plots at Fureneset using a hand-held SKR 1,800 Two-channel Light Sensor (Skye Instruments, Llandrindod Wells, United Kingdom) at 2 m above ground level around midday. NDVI is calculated based on the reflectance in the near infrared (NIR 780–900 nm) and red (660 nm) wavebands using the formula: NDVI = (NIR – Red)/NIR + Red). Values range between 0 and 1 for vegetated surfaces, with high positive values indicating an abundance of photosynthetically active vegetation (Eidenshink, 1992). Measurements were made weekly in field plots in 2013 (3 measurements in November) and from 2 September to 18 December in 2014.

### Determination of Ploidy Level by Flow Cytometry

The ploidy level of all selections was determined by flow cytometry testing of 25 randomly selected plants/selection using a Partec CyFlow Ploidy Analyser (Sysmex Europe GmbH, Norderstedt, Germany). The plants for ploidy testing were sown in a greenhouse in November 2015 and ploidy-tested in December 2015.

### Determination of Freezing Tolerance

This freezing test was undertaken in order to test the efficiency of the two selection methods (FT and CF approaches) in creating divergent freezing tolerance among the selections. Crown segments were used to test the effect of selection on freezing tolerance. Seedlings of all selections were established in trays in the greenhouse at Fureneset in early September 2015 and transferred to outdoor conditions for natural hardening on 3 November. The seedlings were from the same seed lots used for establishing the field trials. Prior to the freezing test at 13 January 2016, the plants were thawed at low temperatures, split into single tillers and trimmed to 5 cm from crown to top and with 1 cm root according to Larsen (1978). Bundles of 10 plants (2 replicates per selection and temperature) were placed in fine humid sand in plastic boxes and left for about 12 h at -2°C to avoid super-cooling of the plants. The boxes were exposed to pre-determined freezing temperatures of between -3°C and -15°C at a cooling rate of 1°C h^-1^ until -10°C was reached and then kept at 3°C h^-1^ until the pre-determined temperature was reached for each treatment, using a programmable freezer in which the temperature was regulated by a monitoring system (Vektek Ltd., Klepp, Norway). The temperature was recorded by a Campbell Scientific CR850 data logger. The plants were thawed at 2°C for 24 h, then transplanted into fertilized soil along with unfrozen controls (two boxes per selection, with the temperature kept at 2°C in darkness as a control) and transferred to a heated greenhouse (15°C, 24 h light) for 28 days. Dry matter regrowth of all individual plants was then measured.

### Climate at the Experimental Sites

**FIGURE 2:**
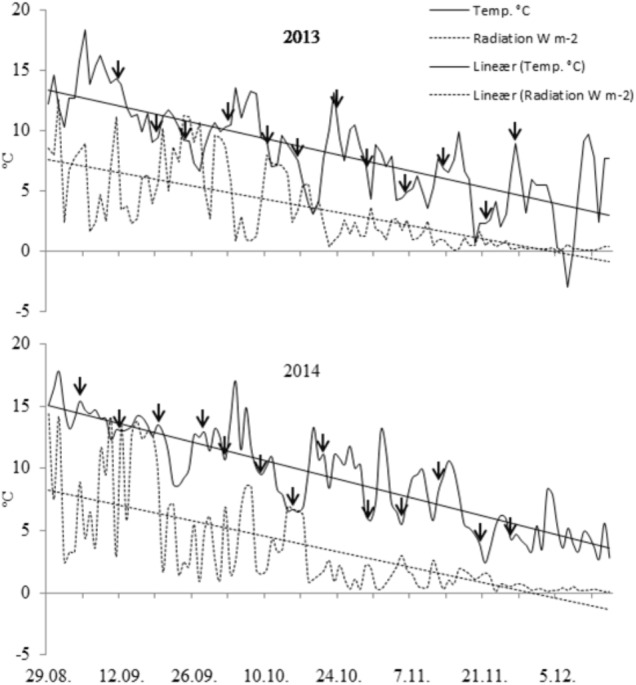
Mean daily air temperature (°C) and radiation (Wm^-2^) at the coastal experimental site (Fureneset) during the period late August-early December in 2013 and 2014. Sampling dates are shown by arrows.

Temperature (°C) and solar radiation (W m^-2^) during the experimental period (September-early December in 2013 and 2014) at Fureneset (on-site weather station) and sampling dates are shown in **Figure 2**. The mean temperature for the 3-month period September-November was 7.4 in 2012, 8.6 in 2013 and 9.9 in 2014. At Bjørke (meteorological station Ilseng), the corresponding temperatures were 4.2 in 2012, 4.6 in 2013, and 6.3 in 2014. For the winter period, December-March the mean temperatures were -0.2 (2012–2013), 4.6 (2013–2014), and 3.7 (2014–2015) at Fureneset, and -8.6 (2012–2013), -0.8 (2013–2014), and -2.8 (2014–2015) at Bjørke. For the standard period 1961–1990 the yearly mean temperature at Fureneset was 7.0°C compared with 3.3°C at Bjørke, and with annual precipitation of 2010 mm and 565 mm at Fureneset and Bjørke, respectively (Norwegian Meteorological Institute, 2017).

### Statistical Analyses

A fully randomized block design was used for both field and laboratory experiments. The effects of the divergent selections were calculated and their statistical significance was tested using GLM (General Linear Model). Broad sense heritability and expected response to selection were estimated using variance component analyses. Normal distribution of the data was checked using the Shapiro-Wilk W test. Tukey’s HSD (Honest Significant Difference) test was used for estimating the significance of differences between means. Linear correlation coefficients (Pearson’s) were calculated for some parameters. Principal Component Analysis (PCA) by eigenvalue decomposition of a data correlation matrix was used for studying field performance of the divergent selections. Values for freezing tolerance were corrected to equal means for boxes within replicates (Aastveit, 1977) and the statistical analyses were performed using AGROBASE generation II (Agronomix Software 2004) or Statistica 12.0PL (Statsoft, Tulsa, OK, United States). Data from field trials, NDVI and the regrowth data from the freezing test were analyzed by multi-factorial analysis using PROC GLM in SAS (SAS 9.4, SAS Institute Inc., Cary, NC, United States). For the analyses of the regrowth data, selections and freezing time were considered as fixed factors.

## Results

### Selection Response

#### Selection Based on Freezing Tests (FT)

The results showed highly significant differences in freezing tolerance between genotypes (*F* = 5.0, *p* < 0.001). The mean value was 4.8, ranging from 1.3 to 7.7 (scale 0–9). Mean value for the selections was 7.0 and 2.6 for the high and low freezing tolerance direction, respectively, with a selection differential of 2.2 in both directions. Broad sense heritability (h^2^_bs_) was 0.78, while response to selection (*R* = S/h^2^) was 1.7 (35%) in both directions. Freezing tests on the first-generation selections (FT-H1 and FT-L1) revealed significant differences in freezing tolerance between genotypes in both selections (*F* = 2.2, *p* < 0.001) with a mean value of 5.6 for FT-H1 and 3.7 for FT-L1 (cfr. Larsen, 1979).

#### Selection Based on Chl-*a* Measurements (CF)

During both years of the selection, differences in PI_ABS_ between genotypes were highly significant (*F* = 12.174, *p* < 0.001 for the first year; *F* = 6.16, *p* < 0.001 for the second year). During the first selection cycle, the mean value was 0.607 (range 0.045–1.378). Mean value for the selections was 0.889 and 0.315 for the high (CF-H1) and low (CF-L1) direction, respectively, which gave a selection differential of 0.287 in both directions. Broad sense heritability (h^2^_bs_) was the same as in the case of freezing tolerance (0.78), whereas the expected response to selection was 0.268 (45%) in both directions.

During the second selection cycle, when the growth conditions were changed to induce depletion of reserves, the mean value was 0.634 (range 0.038–1.301). Mean value for the selections was 0.766 and 0.502 for the high (CF-H2) and low (CF-L2) direction, respectively, which gave a selection differential of 0.132 in both directions. Broad sense heritability (h^2^_bs_) was 0.572, while the expected response to selection (*S* = h^2^ R) was 0.111 (18%) in both directions. In addition, the difference between the progeny of plants selected for high and low PI_ABS_ after freezing during the first year was statistically significant (*P* < 0.01), with a mean value of 0.645 and 0.594 for high and low PI_ABS_ selection, respectively.

### Individual Plant Measurements

#### Leaf Elongation Growth

**FIGURE 3:**
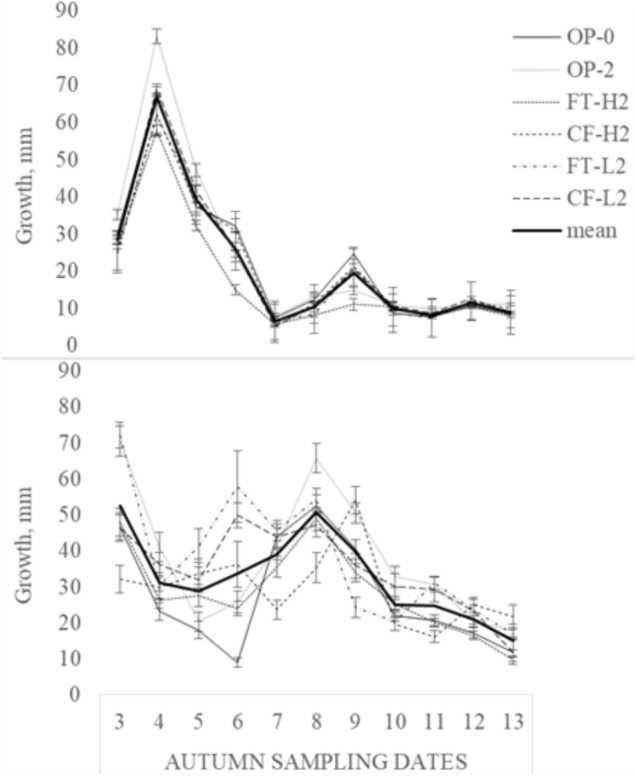
Weekly leaf growth (mm, mean ± SE) averaged over selections in 2013 (upper diagram) and 2014 (lower diagram) during the period 25 September–27 November.

Leaf growth decreased gradually during the autumn 2013, but the response to milder periods in mid- and late October was lower for FT-H2 and OP-2 than for the remaining selections. Averaged over the last 11 samplings, FT-H2 demonstrated the lowest accumulated leaf growth (19.4 mm) and OP-2 the highest (27.2 mm). Leaf elongation was greater during the generally milder autumn of 2014, with an average of 32.5 mm for all selections compared with 23.4 mm for 2013, and the reduced leaf growth appeared later in the experimental period. Accumulated leaf growth, averaged over the last 11 sampling times, varied from 25.9 mm (FT-H1) to 39.6 mm (OP-1) in 2014 (**Figure 3**). Larger variations between the selections were found at the end of the experimental period in 2014 than in 2013 (data not shown). A year × sampling date × selection interaction was found (*F* = 2.11, *p* < 0.001).

#### Developmental Pattern of Selected Photosynthetic Parameters

Average maximum photosynthetic efficiency of PSII (F_v_/F_m_) was 0.82 and 0.81 for 2013 and 2014, respectively. In 2013, an increase in F_v_/F_m_ was observed following a temperature increase in mid-October (sampling dates 6–7) and at the very end of the experimental period. Very small differences were observed between the selections in 2013, whereas in 2014, FT-H2 and FT-L2 formed the extreme entries, with an F_v_/F_m_ value of 0.78 and 0.83, respectively, averaged over the two last sampling times.

Average value of the PI was 1.53 in 2013 and 1.49 in 2014. During the first year there was a weak increase in the PI_ABS_ values, whereas a decrease was observed all through the second autumn, so the mean value over the two last observations was 1.76 and 1.17 in 2013 and 2014, respectively.

### Field Measurements

#### NDVI During Autumn

**FIGURE 4:**
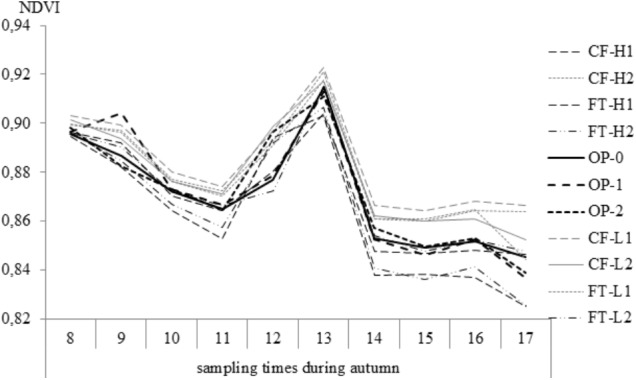
Normalized different vegetation index (NDVI) values during autumn 2014; weekly measurements from 21 October (sampling time 8) to 18 December (sampling time 17) for all selections.

During the 17-week measurement period in 2014, NDVI fell progressively throughout the autumn from the first sampling occasion (0.91) to the 17th (0.85). Increased values were observed following days of increased temperature, such as in early-October (0.94) (17°C on 4 October) and late November (0.91). Sampling dates were significantly different (*F* = 283, *p* < 0.001) as were the selections (*F* = 9.3; *p* < 0.001). Stable values were observed during the last four sampling dates in December, and the NDVI at the last sampling date (17) showed significantly lower value (0.845) than the three earlier samplings (*F* = 8.3, *p* < 0.0001). The lowest values (0.835 and 0.836) were found among the FT-H1 and FT-H2 selections, and the values were significantly lower than for CF-L1 (0.866) and CF-H2 (0.862) (*F* = 15.8, *p* < 0.0001) (**Figure 4**).

During the three measurements of NDVI in November 2013 (data not shown), FT-H2, CF-H1, and FT-L2 demonstrated the lowest NDVI values. During weeks 40–44 in 2013, leaf senescence (yellowing) was observed in FT-H2 and FT-H1 plots and partly in CF-H1 plots, whereas the other plots remained green. Senescence was not observed during the very mild autumn of 2014.

#### Calculated Winter Damage

Estimated winter damage (based on previous autumn performance) averaged over 2 years ranged from 3.6 to 19.5, with the lowest values within the original population. FT-H2 had significantly more winter damage (19.5) than the remaining selections. In the third ley year, however, with no significant differences, more winter damage was observed in plots of FT-H2 (16.7), FT-H1 (11.9), and CF-H1 (8.9) than in the other selections, and with the lowest winter damage (3.7–4.0) in the original populations.

#### Biomass Production Under Diverse Field Conditions: Interactions in Field Trials

Averaged over three ley years, the entries at the Fureneset (coastal) site yielded significantly more than at the Bjørke (continental) site (*F* = 200.0; *p* < 0.001), and the primary (spring) cut was significantly higher at Fureneset (4.46 t DM ha^-1^) than at Bjørke (3.74 t DM ha^-1^). The lowest-yielding selections were FT-H2 (average 3.18 t DM ha^-1^), FT-H1 and CF-H1, which were significantly different from the remaining selections (**Table 1**). At Fureneset, these three selections yielded considerably less than the remaining entries in the primary cut, while at Bjørke FT-H1 and FT-H2 gave significantly lower DMY than the remaining entries. For total DMY (three cuts/year) averaged over 3 years, FT-H2, FT-H1, and CF-H1 were the lowest-yielding selections. The highest DMY in the primary cut (average 4.55 t DM ha^-1^) was observed in OP-0 and OP-1. A significant site × entry interaction (*F* = 10.9; *p* < 0.001) arose, due to different rankings of the selections at the two sites, but the differences were small.

**Table 1:** Dry matter yield (DMY) of three cuts in percentage of total DMY (t ha^-1^) averaged over three ley years at Fureneset (Fu) and Bjørke (Bj) for the original and random mating generations (OP-0, OP-1, and OP-2) and the selections (*n* = 9).

		OP-0	OP-1	OP-2	FT-L1	FT-L2	FT-H1	FT-H2	CF-L1	CF-L2	CF-H1	CF-H2
Fu	DMY1, %	50	52	51	50	51	41	40	49	50	46	51
	DMY2, %	29	28	28	29	29	37	37	30	30	34	30
	DMY3, %	20	19	20	21	20	22	23	21	20	21	20
	DMY, t ha^-1^	9.87	9.97	10.13	9.77	9.21	7.99	7.42	9.31	9.37	8.10	9.44
		^abc^	^ab^	^a^	^abc^	^c^	^de^	^e^	^bc^	^bc^	^d^	^abc^
Bj	DMY1, %	57	56	55	55	55	58	56	56	55	56	58
	DMY2, %	24	25	26	26	26	24	25	25	26	25	24
	DMY3, %	19	19	19	20	20	19	19	19	20	19	18
	DMY, t ha^-1^	7.22	6.89	6.81	6.77	6.64	6.13	6.00	6.71	6.61	6.66	6.61
		^a^	^ab^	^ab^	^ab^	^b^	^c^	^c^	^b^	^b^	^b^	^b^

*Different letters (a–e) within rows indicate significant differences between entries.*

### Selection Pattern Over Two Selection Cycles

**FIGURE 5:**
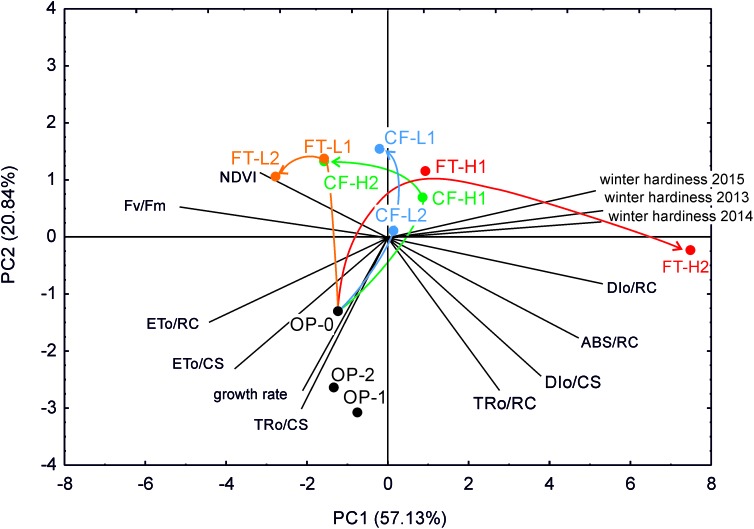
Biplot of principial component analysis (PCA) for winter survival, leaf growth rate, NDVI and chlorophyll fluorescence parameters before winter (means for all measurement years). Selection groups are indicated and named as in **Figure 1**. Selection driven changes in the relationships between traits are indicated with arrows. Please note that CF-H2 (CF-L2) selection differed in the conditions before hardening to frost from CF-H1 (CF-L1), which changed the direction of selection from the similar of FT.

The first-generation selections toward high freezing tolerance behaved very similarly and for both FT-H1 and CF-H1 in the direction of improved winter hardiness (**Figure 5**). However, after the second generation of high freezing tolerance selection, the two groups diverged. FT-H2 separated from the rest and correlated with winter damage, whereas CF-H2 performance correlated with NDVI. NDVI depends on the chlorophyll content and plant coverage in autumn, which in turn is determined on winter damage in the previous winter. At the end of the experimental period in both years (mean of the last two sampling occasions), PI_ABS_ and NDVI were negatively correlated with winter damage (**Figure 5**). Selection in the low direction caused smaller differences, and both CF and FT selections were correlated with NDVI. In the three generations of the original population, there were some minor changes during the selection cycles (seed multiplication). Natural selection under field conditions from the first to the second year affected only FT-H2, due to increasing winter damage and decreasing PI_ABS_/PI_CSm_. Similar changes were not observed for the remaining selections. Similarly, when only analyzing the last two sampling occasions in both years, FT-H2 differed from the remaining groups by being positively correlated with winter damage and mean growth and negatively correlated with PI, F_v_/F_m_, and ET_o_/CS. Moreover, NDVI was positively correlated with PI_ABS_.

### Trait Correlations

There was strong negative correlations between winter damage and F_v_/F_m_, ψ_o_, φ_Eo_, ET_o_/RC, and DI_o_/RC (all below -0.80), and between NDVI and winter damage, in 2013 and in 2015. Mean leaf growth was negatively correlated with winter damage and slightly positively correlated with NDVI (**Table 2**).

**Table 2:** Simple correlations between mean winter damage and NDVI for the sampling dates (sd) 14–17 (at the end of the growing season) and chlorophyll *a* (Chl-*a*) fluorescence parameters during two experimental seasons.

Parameters	Mean winter damage	NDVI
F_v_/F_m_	-0.83	0.34
ABS/RC	0.73	-0.42
ψ_o_	-0.80	0.61
φ_Eo_	-0.83	0.53
PI_CSo_	-0.76	0.22
PI_CSm_	-0.64	0.19
PI_ABS_	-0.59	0.40
TR_o_/CS	-0.45	-0.25
ET_o_/CS	-0.69	-0.02
DI_o_/CS	0.57	-0.51
ET_o_/RC	-0.80	0.47
TR_o_/RC	0.34	-0.32
DI_o_/RC	0.88	-0.42
Winter damage 2013	0.92	-0.63
Winter damage 2014	0.98	-0.52
Winter damage 2015	0.94	-0.71
Mean winter damage	-	-0.60
NDVI sd 14-17	-0.60	-
Mean leaf growth	-0.40	0.17

### Verification of Ploidy Levels and Freezing Tolerance (Regrowth Capacity After Freezing)

On testing the freezing tolerance of all selections, the percentage of live plants and dry matter regrowth confirmed the freezing tolerance in the high selection groups, with clear differences compared with the freezing-sensitive selections (**Table 3**). Tests on the ploidy level of the selections revealed that it changed during the selection cycles. In addition, it differed between the selection methods. In the original populations (OP), the frequency of tetraploids increased with the generations of random mating. Selection in the direction of high freezing tolerance using the whole plant freezing test gave completely diploid populations (FT-H1 and FT-H2), while selection for low freezing tolerance gave completely tetraploid populations (FT-L1 and FT-L2). The first-generation selection by the Chl-*a* fluorimetry method in the direction of high freezing tolerance (CF-H1) was completely diploid, whereas mixed ploidy was observed in the second generation (CF-H2). In the low direction (CF-L), the frequency of tetraploids increased from the first to the second generation.

**Table 3:** Percentage of diploid (2n) and tetraploid (4n) plants and effects of frost exposure.

	Ploidy		Frost exposure		
	% of pop.		No. of alive plants, % of pop.	Regrowth DM, g	DM plant^-1^, g
Pop./selection group	2n	4n	
OP-0	55	45	6.3^ef^	0.034^c^	0.017^cd^
OP-1	32	68	16.4^de^	0.122^bc^	0.060^ab^
OP-2	16	84	4.4^ef^	0.010^c^	0.009^cd^
FT-H1	100	0	40.6^ab^	0.288^b^	0.071^a^
CF-H1	100	0	45.7^a^	0.441^a^	0.078^a^
FT-H2	100	0	46.8^a^	0.241^b^	0.049^abc^
CF-H2	54	46	33.1^bc^	0.239^b^	0.064^ab^
FT-L1	0	100	5.1^ef^	0.024^c^	0.022^bcd^
CF-L1	92	8	24.7^cd^	0.125^bc^	0.042^abcd^
FT-L2	0	100	0.0^f^	0.000^c^	0.000^d^
CF-L2	65	35	7.2^ef^	0.038^c^	0.026^bcd^
Mean			20.93	0.142	0.67
*R*^2^			0.88	0.76	0.040

*No. of alive plants (% of pop.), regrowth dry matter (DM) and dry matter plant**^-^**^1^ in the populations and selection groups. Mean values and R^2^ are added to the frost exposure results (n = 16). Different letters (a–f) within columns indicate significant differences between entries*.

## Discussion

Freezing tests on whole plants (FT) and chlorophyll *a* fluorimetry on frozen detached leaves (CF) as a selection tool for freezing tolerance and winter hardiness, or selection efficiency in general, proved successful. Both methods revealed ample genetic variation for freezing tolerance in the original ×*Festulolium* population (FuRs0357) and were able to establish divergent selections with significantly different freezing tolerance. Differences in winter hardiness were apparent in selections for both high and low freezing tolerance and differed between generations.

During the first cycle of selection for high freezing tolerance, the performance of the FT and CF selections was positively correlated with winter hardiness or high freezing tolerance, which again was connected with early growth cessation (**Figure 5**). The realized heritability was similar for the FT and CF selections following the first selection cycle, indicating the same genetic and environmental variation within and between selections. A similar pattern was therefore expected in the second selection cycle and this was the case for the FT-H2 selection, which displayed improved winter hardiness and earlier growth cessation. However, a different response (selection of both photosynthetically active in cold and freezing tolerant, instead of freezing tolerant plants only) was observed for the CF-H2 selection, probably due to the starvation imposed on the plants before the second selection cycle, resulting in a lower selection response (18%) than expected (45%). This led to selection toward higher photosynthetic performance and higher NDVI, which resulted in the selection of plants with high photosynthetic activity under cold. These plants cease growth later. We speculate that plants which ceased growth early were more susceptible to freezing during the second cycle of CF selection due to dark/moderate temperature treatment preceding cold acclimation which simulated the conditions of warmer late autumns/early winters at high latitudes, irrespective on unchanged conditions of cold acclimation compared to the first round of the selection (plants were cold acclimated in the light). The same conditions of cold acclimation led us to pay attention to the preceding period. It may affect the effectiveness of cold acclimation due to low photosynthetic activity observed in plants characterized with early growth cessation in autumn connected with the possible depletion of assimilate reserves during the dark period. Such a stress-effect of depletion of assimilate reserves before winter on freezing tolerance of plants with limited photosynthetic activity during cold acclimation has only been reported previously in trees (Galvez et al., 2013). Alternative explanations are also possible.

During the field trials, the FT-H2 selection suffered severely from winter damage. This could be due to too intense selection creating a population with high frost tolerance but with narrow adaptation, which suffered during the relatively mild autumns of the experimental years. One generation of selection using freezing tests may therefore be sufficient in selection of non-native species in Norway such as *L. perenne* and ×*Festulolium*. In addition, the reduction in number of plants for selection in the second cycle, combined with the same selection intensity (10%), might have led to inbreeding depression, and possibly augmented the effects of mixed ploidy level. Differences in selection response for freezing tolerance depending on adaptation have also been observed in *Dactylis glomerata*, with cultivars of Danish (southern) origin showing a higher response to selection than cultivars adapted to northern Norway, both with selection intensity of 5% (Larsen, 1994). In our study selection of winterhardy plants based on freezing tests on whole plants (FT) have a similar effect as selection based on chlorophyll fluorescence measurements after freezing of detached leaves. This was evident only after the first cycle of selection, because during the second cycle the conditions preceding cold acclimation were considerably different during CF and FT selection.

Cold acclimation of cold-tolerant herbaceous plants requires energy, and the plants must maintain photosynthesis as well as avoiding photoinhibition (see Rapacz et al., 2004). It has been shown that freezing tolerance of grasses like *Phleum pratense* and *L. perenne* will be reduced when cold acclimation takes place at higher temperatures under shorter photoperiods with low light intensity; a scenario expected in northern regions with climate change (Dalmannsdottir et al., 2017). Active leaf growth in *L. perenne* and ×*Festulolium* even in December has been reported by Østrem et al. (2014), demonstrating that these species still grow under low-light conditions in late autumn and may suffer from light starvation when the growing seasons become longer in the Nordic region due to climate changes. Without a reasonable level of photosynthetic activity in autumn, these plants would not be able to accumulate reserves ensuring winter survival (Savitch et al., 2002). The early growth cessation observed in the FT-H plots was also reflected in visible senescence of the plants during autumn 2012 in which the mean temperature of the period September – October was 8.2°C compared with 9.3°C for the standard period 1961–1990 and 10.1°C as mean for the period 1991–2017. This also correlates with the low NDVI values from late November 2013 in the FT-H2 population, which was supported by the lower DMY. The mild autumn of 2014 probably affected growth development pattern, since the discrimination between the cultivars only became clear very late in autumn. Natural selection on the plots during the experimental period may also have affected the results. Our hypothesis that selection for high freezing tolerance using direct freezing tests (FT approach) will lead to earlier growth cessation compared with selection based on chlorophyll fluorescence (CF approach) was partly supported.

In general, a negative correlation between freezing tolerance and productivity has been reported for forage grasses (Hides, 1979). This partly explains the lower yield capacity of the diploid selections FT-H1, FT-H2, and CF-H1 observed at both sites. At the milder Fureneset site, the observed reduction in productivity is most likely related to low photosynthetic activity as demonstrated by low winter survival and considerably lower DMY in the primary cut, which for FT-H2 was especially low in the second ley year. At Bjørke, lower total DMY was observed in these selections, but with no differences in the primary cut and no indications of a strong impact of the previous autumn and winter. These results indicate that the selection intensity applied to the originally non-adapted FuRs0357 population might have been too high, so that the effect of ploidy became the dominant factor affecting the performance of the selections since in general diploids are yielding less than tetraploids (Helgadóttir et al., 2018). The hypothesis that direct selection (FT) correlates better to field conditions than the indirect CF selection method was not supported, since the results were not solely due to selection.

Chlorophyll fluorescence parameters measured in autumn revealed the main differences between the FT and CF selections. Plants of the FT-H2 population were distinct from plants of other selections by having lower PSII photochemical activity, which suggests lower photosynthetic activity in autumn (Strasser et al., 2000). Winter survival was negatively correlated with PSII performance indices such as PI (PI_ABS_, PI_csm_, PI_cso_), maximum photochemical efficiency of PSII (F_v_/F_m_) and an energy flux for electron transport in PSII (ET_o_/CS). This negative correlation might also be related to early growth cessation, although growth cessation was not connected with photosynthetic activity and seemed neutral relative to winter survival. The reduced winter survival and photosynthetic activity in autumn observed in the FT-H2 selection questions the importance of freezing tolerance for winter survival at the experimental site (Fureneset). Although the winter period at that site is not very harsh, with a mean temperature of 1.7°C for the period December-February (standard period 1961–1990), freezing tolerance is still an important trait for survival, and plants should be well balanced between freezing tolerance and growth cessation. The winter conditions are characterized by varying temperature, e.g., with the lowest and highest mean monthly temperature during the period 1991–2017 of 9.6°C (-2.9 to 6.7°C) for December and similarly 7.0°C (-2.6 to 4.4°C) for January. Imbalances between adaptation area and actual growing area for grass cultivars have been observed when testing winter-hardy, northern-adapted cultivars of indigenous or well-adapted species in southern Norway. In southern lowland areas (59–62°N), cultivars adapted to northern regions (65–67°N) cease growth earlier than southern-adapted cultivars. Reserves for maintenance may be used instead of accumulating carbohydrate reserves during autumn, resulting in low survival rate of e.g., *F. pratensis* in the next year, although the inherent winter hardiness of these cultivars is normally very high (Nesheim and Langerud, 2014). Several studies have shown correlations between changes in non-structural carbohydrates, mainly the low-molecular fructans, and winter survival due to the effects of fructans on membrane stability (see Hisano et al., 2004; Livingston et al., 2009). Tests of *P. pratense* and *L. perenne* using whole plants have shown that the fructan content during winter correlates with freezing tolerance, suggesting a more efficient carbohydrate metabolism in plants capable of tolerating harsh winter conditions (Østrem et al., 2011).

The CF-H2 selection showed a positive correlation between photochemical performance in autumn and winter survival. This might be an effect of selection of plants able to cold-acclimate just after depletion of reserves, i.e., plants with higher photosynthetic activity in autumn. This trait seems to be more important for winter survival at the coastal site than freezing tolerance. The CF-H2 selection also showed the highest potential for DMY of all selections in the direction of high freezing tolerance.

Selection toward low freezing tolerance (both FT and CF) mainly progressed in the direction toward higher photosynthetic activity (NDVI and PI_ABS_) and with smaller changes between generations for both selection methods. As shown by Larsen (1985) in populations of *D. glomerata*, selection for low freezing tolerance did not give as consistent effects as selection for high freezing tolerance. The non-selected intercrossed original population remained a separate group with smaller changes over generations due to natural selection during seed propagation.

Freezing tolerance was confirmed in all high-direction selections (**Table 3**). The resultant divergent selections are therefore products of the joint effect of selection method, mixed ploidy level and changes in morphological and physiological characters of the plants due to selection. Earlier studies have shown that diploid *L. perenne* cultivars of Nordic origin have better freezing tolerance than tetraploid cultivars (Yamashita and Shimamoto, 1996; Sugiyama, 1998). Recent studies of freezing tolerance in 154 *L. perenne* genebank accessions of wide geographical origin and 22 European cultivars have also consistently demonstrated the difference between diploid and tetraploid plants for this trait (Aleliūnas and Brazauskas, 2018; Helgadóttir et al., 2018). This was confirmed in the present study; the FT selection method consistently gave pure diploid and tetraploid populations following selection for high and low freezing tolerance, respectively, over the two selection cycles. The CF method gave the same result only for selection for high freezing tolerance in the first selection cycle. Selection in the direction of low freezing tolerance gave mixed ploidy (92/8% 2n/4n in CF-L1), but the frequency of tetraploid genotypes increased as expected during the second selection cycle (CF-L2). The mixed ploidy (54/46% 2n/4n in CF-H2) is very difficult to explain, since the CF-H1 population was 100% diploid.

Changes in genomic constitution during selection and seed propagation have also been observed in earlier studies of the FuRs0357 population, where both diploid and tetraploid plants have been found (Bartoš et al., 2011). The genetic irregularities observed in the present study demonstrate some of the problems of introgression breeding, as observed previously at the phenotypic level (Østrem et al., 2007) and genetic level (Lideikytė et al., 2006; Bartoš et al., 2011). Controlling the transfer of e.g., stress tolerance genes in an introgression breeding program on the diploid level is challenging. Although genetic drift and progressive loss of fescue chromosomes over generations have been observed for amphitetraploids (Zwierzykowski et al., 2006; Ghesquière et al., 2010), this approach seems to generate more genetically stable populations than the introgression breeding program generating diploid *Lolium* populations with small *Festuca* segments following backcrossing to *Lolium.*

The positive correlation between photochemical performance in autumn and winter survival expressed in the CF-H2 selection may be the most favorable combination of traits under expected future winter conditions in coastal southern locations in Norway, where active photosynthesis during warmer autumns before snowfall and freezing may be critical for overwintering. Plants with early growth cessation and reduced photosynthetic activity will be less winter-hardy which may be connected with increased respiration rate and depletion of reserves during the expected warmer autumns and winters. This applies mainly to non-adapted species like *L. perenne* and ×*Festulolium*, but may also be a useful strategy for breeding in other species under future climate conditions.

## Conclusion

Freezing tests were considered as the best way to select more winterhardy forage grasses due to increased winter survival rate of freezing tolerant plants. Warmer autumns and winters observed during recent decades in Norway have however, revealed that higher winter survival rates and yielding of plants depended not only on good freezing tolerance but also on high photosynthetic activity before winter. Although both approaches of selection tested in this study proved successful for developing freezing-tolerant plants, the selection method where the results of winter damages are directly connected with photosynthetic activity during cold acclimation might be better fitted to the current needs of plant breeding. In the present study we showed that chlorophyll *a* fluorescence measurements taken after freezing of leaves from plants cold-acclimated after depletion of reserves appear as one good option for selection of winterhardy and well performing populations of ×*Festulolium*. Freezing tests might still be a valuable option also for non-native species, but the selection intensity has to be closely adapted to the climate conditions of the growing area to ensure plants with sufficient photosynthetic activity during autumn enabling the plants for accumulation of reserves for good winter survival and production capacity over years.

## Author Contributions

AL, PM, OR, and MR planned and designed the selection study. AL and MR performed the selections. PM and OR organized the seed propagations. LØ and MR planned and designed the field trials and measurements on individual plants. LØ validated the frost tolerance. MR, AL, and LØ analyzed the data. LØ, MR, and OR wrote the manuscript.

## Conflict of Interest Statement

The authors declare that the research was conducted in the absence of any commercial or financial relationships that could be construed as a potential conflict of interest.
